# Highly Sensitive 3D‐Nanoplasmonic‐Based Epidermal Growth Factor Receptor Mutation Multiplex Assay Chip for Liquid Biopsy

**DOI:** 10.1002/smsc.202400101

**Published:** 2024-06-26

**Authors:** Ji Young Lee, Byeong‐Ho Jeong, Ho Sang Jung, Taejoon Kang, Yeonkyung Park, Jin Kyung Rho, Sung‐Gyu Park, Min‐Young Lee

**Affiliations:** ^1^ Advanced Bio and Healthcare Materials Research Division Korea Institute of Materials Science (KIMS) 797, Changwon‐daero, Seongsan‐gu Changwon‐si Gyeongsangnam‐do 51508 Republic of Korea; ^2^ Division of Pulmonary and Critical Care Medicine Department of Medicine Samsung Medical Center SungkyunKwan University School of Medicine 81, Irwon‐ro, Gangnam‐gu Seoul 06351 Republic of Korea; ^3^ Bionanotechnology Research Center Korea Research Institute of Bioscience and Biotechnology (KRIBB) 125 Gwahak‐ro, Yuseong‐gu Daejeon 34141 Republic of Korea; ^4^ School of Pharmacy Sungkyunkwan University 2066 Seobu‐ro, Jangan‐gu Suwon, Gyeonggi‐do 16419 Republic of Korea; ^5^ Department of Convergence Medicine Asan Medical Center College of Medicine University of Ulsan Olympic‐ro 43‐gil, Songpa‐gu Seoul 05505 Republic of Korea

**Keywords:** epidermal growth factor receptor mutations, liquid biopsies, multiplex diagnostics, nanoplasmonics, wild‐type inhibitors

## Abstract

Economical mutation detection method with high analytical and clinical sensitivity is necessary for early cancer diagnosis and screening. In this study, a novel 3D‐nanoplasmonic‐based multiplex mutation assay chip is developed to detect epidermal growth factor receptor (EGFR) mutations. This assay kit comprises a 3D‐nanoplasmonic substrate immobilized with capture probes and primer–probe sets for recombinase polymerase amplification, wild‐type inhibition, and fluorescence detection, enabling multiplex detection of EGFR exon 19 deletions, exon 20 insertions, and exon 21 L858R point mutations. The strategy facilitates the detection of all deletions and insertions within the target region with extremely high analytical sensitivity, detecting as low as 1 × 10^−9^% mutation frequency, implying three copies/reactions and 100 zM. The synergistic effects of plasmon‐enhanced fluorescence from the 3D‐nanoplasmonic substrate and wild‐type inhibitor contribute to this high analytical sensitivity. Moreover, the developed chip exhibits 100% accuracy in the clinical testing of plasma samples from normal individuals and patients with benign lung tumor and malignant lung tumor. With high sensitivity and multiplexing capabilities, this assay operates at a low reaction temperature (around 37 °C) and requires a short processing time, ≈70 min post‐cell‐free DNA extraction. These features make the chip a valuable tool for easy and widespread cancer screening.

## Graphical abstract

An epidermal growth factor receptor (EGFR) mutation multiplex assay chip is developed by integrating a 3D‐nanoplasmonic microarray, recombinase polymerase amplification and a wild‐type inhibitor for liquid biopsy. This assay chip demonstrates remarkably high analytical sensitivity owing to the synergistic effects of plasmonic substrate‐enhanced fluorescence and wild‐type inhibitor. It exhibits high accuracy in detecting circulating tumor DNA in lung cancer patients.

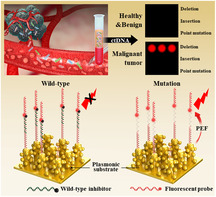

## 1 Introduction

Epidermal growth factor receptor (EGFR) mutations are key biomarkers for cancer, particularly lung cancer.^[^ ^1^, ^2^, ^3^ ^]^ It has been reported that EGFR mutations were observed in over 50% of patients with lung cancer, with EGFR exon 19 deletions (E19dels), exon 21 (E21) L858R point mutations, and exon 20 insertions (E20ins) accounting for ≈50%, 40%, and 5%, respectively.^[^ ^4^, ^5^, ^6^ ^]^ However, these estimates are based on direct or next‐generation sequencing (NGS) analyses that exhibit low sensitivity; therefore, the results may not be accurate.^[^ ^7^ ^]^ The 5 year survival rates for lung cancer are ≈80% for stage 1, 60% for stage 2, 30% for stage 3, and 20% for stage 4. This emphasizes that earlier diagnosis and treatment of lung cancer correlate with higher survival rates.^[^ ^8^ ^]^ Accurate and highly sensitive detection of EGFR mutations is paramount for early diagnosis of lung cancer, as well as for monitoring the efficacy of EGFR‐targeted therapeutics and detecting cancer recurrence.^[^ ^9^ ^]^

Liquid biopsy using circulating tumor DNA (ctDNA) is a less invasive and patient‐friendly approach for cancer screening, offering an alternative to traditional surgical tumor biopsies.^[^ ^10^ ^]^ NGS technology has led to a significant transformation in genomics research. However, several challenges, including low mutation frequency sensitivity (≈1%), high cost, prolonged turnaround time (≈9 days for liquid biopsy), and need of specialized equipment, hinder its widespread application for cancer screening and treatment.^[^ ^11^ ^]^ Compared to NGS, mutation‐targeted real‐time polymerase chain reaction (PCR) and droplet digital PCR technologies facilitate rapid detection with enhanced mutation frequency sensitivity (≈0.1%–0.01%).^[^ ^12^ ^]^ However, these approaches still exhibit low analytical sensitivity and do not account for the extensive diversity in mutations among individuals.^[^ ^13^ ^]^ For example, E19del usually occurs within a 25‐base‐pair range, resulting in 2^25^ possible deletion mutation cases.^[^ ^14^ ^]^ However, the U.S. Food and Drug Administration–approved EGFR mutation test kits, such as the cobas EGFR Mutation Test and the Qiagen therascreen EGFR RGQ PCR Kit, only detect the most common 29 and 14 deletion mutations using each deletion target primer, respectively.^[^ ^10^, ^12^, ^15^, ^16^, ^17^ ^]^ Therefore, these methodologies fail to detect many deletion mutations, leading to low clinical sensitivity.^[^ ^18^ ^]^ Currently, NGS‐ and PCR‐based ctDNA mutation analysis methods have not received clinical FDA approval for cancer screening; they have only been FDA approved for companion diagnostics for targeted therapy in advanced cancer patients.^[^ ^19^ ^]^ Effective early cancer diagnosis and monitoring via ctDNA detection requires methods with extremely high analytical and clinical sensitivity that account for the extensive diversity in mutations.^[^ ^19^, ^20^ ^]^

In this study, we developed a highly sensitive EGFR mutation multiplex assay chip consisting of 3D‐nanoplasmonic microarray pre‐immobilized capture probes, primers for recombinase polymerase amplification (RPA) that simultaneously amplified the wild‐type and mutated DNAs, and probes for wild‐type inhibition and fluorescence signal detection. The 3D plasmonic nanostructures, composed of densely arranged gold (Au) nanopillars decorated with Au nanoparticles, were prepared as reported in our previous work.^[^ ^21^ ^]^ In that study, we investigated the plasmon‐enhanced fluorescence (PEF) effect for highly sensitive protein detection. Protein detection typically involves detecting only one target protein using one antibody. In contrast, ctDNA detection demands technology capable of detecting various mutations in the target gene region, with high mutation frequency sensitivity compared to wild‐type sequences. In this study, to comprehensively detect ctDNA with high mutation frequency sensitivity, we integrated PEF technology with newly designed primer techniques using wild‐type inhibitors to enhance various ctDNA signals while suppressing the wild‐type signals. The 3D nanostructures exhibit a robust PEF effect even for a fluorescence dye positioned at a distance of 20–30 nm (≈equivalent to DNA 60–88 base pairs [bp]) from the surface owing to enhanced light–matter interactions within the 3D space. Moreover, the quencher‐labeled inhibitor specific to the wild type not only quench the fluorescence emanating from wild‐type DNA but also significantly impedes the amplification of wild‐type DNA. We evaluated the analytical sensitivity and specificity of the 3D‐nanoplasmonic‐based EGFR mutation multiplex assay chip for the detection of E19dels, E20ins, and E21 L858R point mutations. We compared the clinical sensitivity of our method with that of NGS using cfDNA extracted from plasma samples of patients with malignant lung tumors at various stages (stages 1–4). Furthermore, clinical specificity of our method was evaluated using cfDNA extracted from plasma samples of patients with benign lung tumors and normal individuals (tumor‐free). By comparing our method with existing approaches, it was confirmed that the developed chip exhibited superior analytical and clinical sensitivity. Therefore, our chip presents an innovative and economical strategy for the highly sensitive and rapid multiplex detection of genetic mutations, facilitating early cancer diagnosis and monitoring.

## 2 Results and Discussion

### 2.1 Design of 3D‐Nanoplasmonic‐Based EGFR Mutation Multiplex Assay Chip

**Figure 1:**
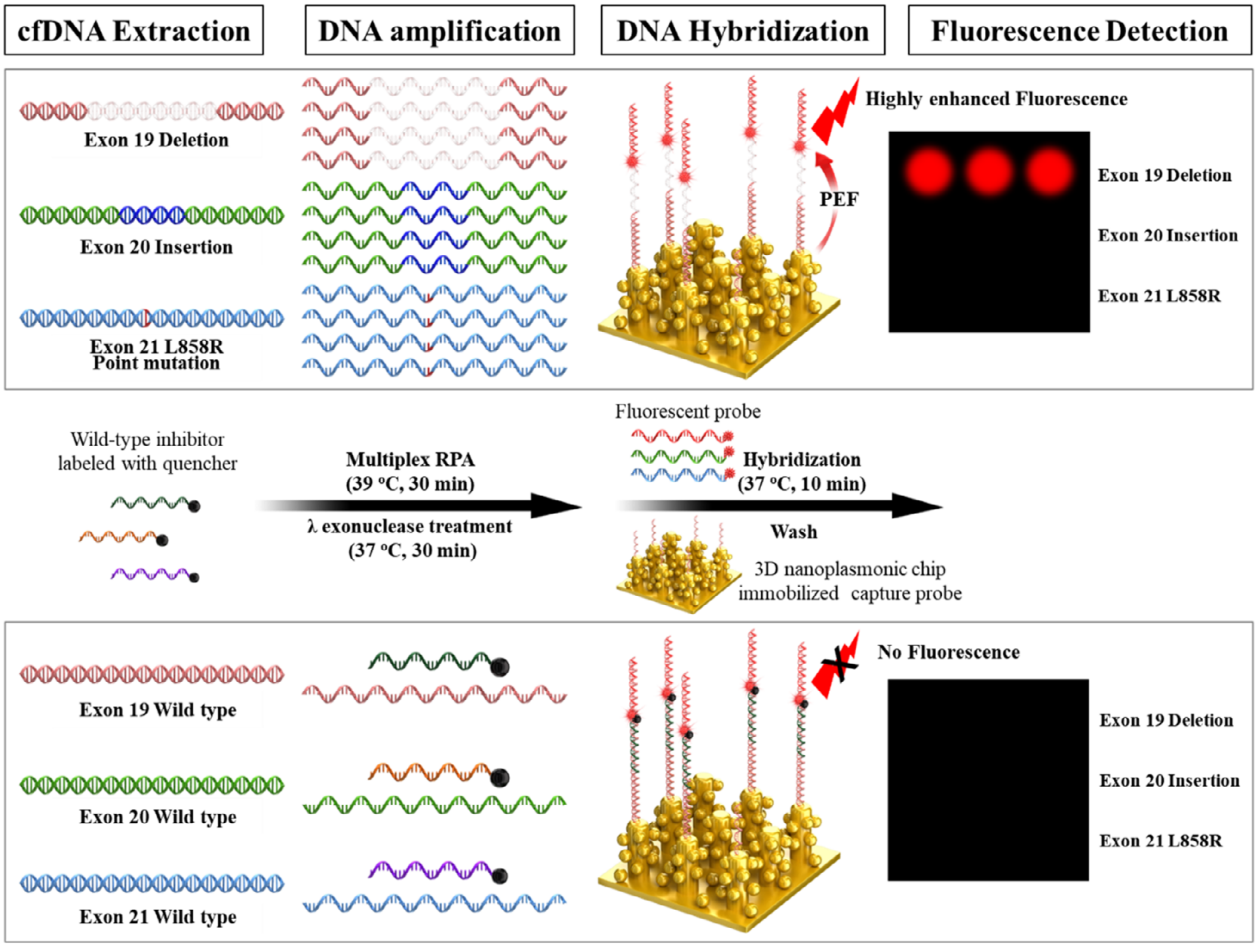
Scheme of the 3D‐nanoplasmonic‐based epidermal growth factor receptor (EGFR) mutation multiplex assay chip.

**Figure** **1** illustrates flow diagram for highly sensitive multiplex ctDNA detection using the 3D‐nanoplasmonic microarray capable of simultaneously detecting E19dels, E20ins, and E21 L858R point mutations. We designed primers for the isothermal amplification of the RPA and wild‐type inhibitors, fluorescent probes, and capture probes (**Table** **1**). To detect E19del or E20ins mutations, primer sequences identical to those of the wild‐type (non‐mutant), excluding regions prone to E19del or E20ins, were used. These primers allowed the amplification of both the wild‐type and mutated genes. In contrast, to detect E21 L858R point mutation, the forward primer had its 3′‐end sequence matched to the point mutation, enabling specific amplification of the point‐mutated gene. The 5′ ends of all reverse primers were phosphorylated. The wild‐type inhibitor sequence was identical to that of the wild‐type region prone to E19del, E20ins, or E21 L858R point mutation and labeled with quenchers at its 3′ ends. These wild‐type inhibitors can only hybridize to the wild‐type, not the mutated genes, resulting in inhibition of amplification and quenching of fluorescence in wild‐type DNA. The sequence of the fluorescent probe was complementary to that of the forward primer and labeled with cyanine dye (Cy5) at the 3′ end. The sequence of the capture probe was identical to that of the reverse primer and labeled with biotin at the 5′ end. These fluorescent and capture probes can hybridize to both the wild‐type and mutant genes.

**Table 1:** Sequences of the templates, primers, and probes used in this study. The visibility of labeled nucleic acid probes, bold formatting serves little significant purpose.

Target gene	Oligo type		Sequence (5′–3′)	Size [nt]
EGFR E19del	Template	Wild‐type	GTGAGAAAGTTAAAATTCCCGTCGCTATCAAGGAATTAAGAGAAGCAACATCTCCGAAAGCCAACAAGGAAATCCTCGATGTGAGTTTCTGCTTTGCTGT	100
		Deletion (c.2240_2251del12)	GTGAGAAAGTTAAAATTCCCGTCGCTATCAAGGAATCATCTCCGAAAGCCAACAAGGAAATCCTCGATGTGAGTTTCTGCTTTGCTGT	88
	Primers and probes	Forward primer	GTGAGAAAGTTAAAATTCCCGTCGCTATCAAG	32
		Reverse primer	P‐ACAGCAAAGCAGAAACTCACATCGAGGATT	30
		Wild‐type inhibitor	TTCGGAGATGTTGCTTCTCTTAATTC‐**BHQ2**	26
		Fluorescent probe	**Cy5‐**CTTGATAGCGACGGGAATTTTAACTTTCTCAC	32
		Capture probe	**Biotin**‐ACAGCAAAGCAGAAACTCACATCGAGGATT	30
EGFR E20ins	Template	Wild‐type	CACTGACGTGCCTCTCCCTCCCTCCAGGAAGCCTACGTGATGGCCAGCGTGGACAACCCCCACGTGTGCCGCCTGCTGGGCATCTGCCTCAC	92
		Insertion (c.2300_2309 dupAGCGTCGAC)	CACTGACGTGCCTCTCCCTCCCTCCAGGAAGCCTACGTGATGGCCAGCGTGGACAGCGTGGACAACCCCCACGTGTGCCGCCTGCTGGGCATCTGCCTCAC	101
	Primers and probes	Forward primer	CACTGACGTGCCTCTCCCTCCCTC	24
		Reverse primer	P‐GTGAGGCAGATGCCCAGCAGGCG	23
		Wild‐type inhibitor	GCACACGTGGGGGTTGTCCACGCTGGCCATCACGTAGGCTTCCTG‐**BHQ2**	45
		Fluorescent probe	**Cy5‐**GAGGGAGGGAGAGGCACGTCAGTG	24
		Capture probe	**Biotin**‐GTGAGGCAGATGCCCAGCAGGCG	23
EGFR E21 L858R	Template	Wild‐type	CGCAGCATGTCAAGATCACAGATTTTGGGCTGGCCAAACTGCTGGGTGCGGAAGAGAAAGAATACCATGCAGAAGGAGGCAAAGTAAGGAGGTGGCTTTAGGT	103
		L858R mutation (c.2573 T > G)	CGCAGCATGTCAAGATCACAGATTTTGGGCGGGCCAAACTGCTGGGTGCGGAAGAGAAAGAATACCATGCAGAAGGAGGCAAAGTAAGGAGGTGGCTTTAGGT	103
	Primers and probes	Forward primer	CGCAGCATGTCAAGATCACAGATTTTGGGCG	31
		Reverse primer	P‐ACCTAAAGCCACCTCCTTACTTTGCCTCCTTC	32
		Wild‐type inhibitor	CCAGC‐**BHQ2**	5
		Fluorescent probe	**Cy5‐**CCCGC	5
		Capture probe	Biotin‐ACCTAAAGCCACCTCCTTACTTTGCCTCCTTC	32

For the assay, multiplex RPA, a rapid isothermal amplification method, is performed for 30 min at 39 °C using extracted cfDNA, designed primers, and wild‐type inhibitors. This can increase the amplification ratio of the mutations compared to that of the wild‐type due to the presence of wild‐type inhibitors. Subsequently, for efficient hybridization, the phosphorylated strands of RPA products are enzymatically degraded using lambda exonuclease, resulting in single‐stranded DNA (ssDNA). Afterward, the ssDNA, fluorescent probe, and an additional wild‐type inhibitor are hybridized to the 3D‐nanoplasmonic chip pre‐immobilized with the capture probes. The amplicons from mutated DNAs on the nanoplasmonic chip exhibit enhanced fluorescence signals due to the PEF effect, whereas the amplicons from wild‐type DNAs cannot show fluorescence signal due to the presence of the quencher‐labeled wild‐type inhibitor. The type of EGFR mutation is identified based on the position‐specific fluorescence signals. Our strategy, utilizing the PEF effect on the nanoplasmonic substrate and wild‐type inhibitor, significantly enhances clinical sensitivity by detecting all deletions and insertions in the target region, as well as analytical sensitivity.

### 2.2 PEF Effect of the 3D‐Nanoplasmonic Substrate on Mutated DNA

In this study, we used our previously developed 3D‐nanoplasmonic substrate.^[^ ^21^ ^]^ The substrate was composed of arranged Au nanopillars densely decorated with AuNPs on the PET film, as shown in the scanning electron microscopy images (Figure S1, Supporting Information). The fabrication method and physical mechanism involved in the formation of high‐density spherical AuNPs on the Au nanopillars have been previously described.^[^ ^21^ ^]^ To immobilize the capture probes on the nanoplasmonic substrate, a mixture of streptavidin and an excess of biotinylated capture probe was directly microspotted onto the nanoplasmonic substrate through a one‐step process. The remaining areas of the substrate were coated with bovine serum albumin (BSA) to inhibit nonspecific binding, thereby enhancing specificity.

**Figure 2:**
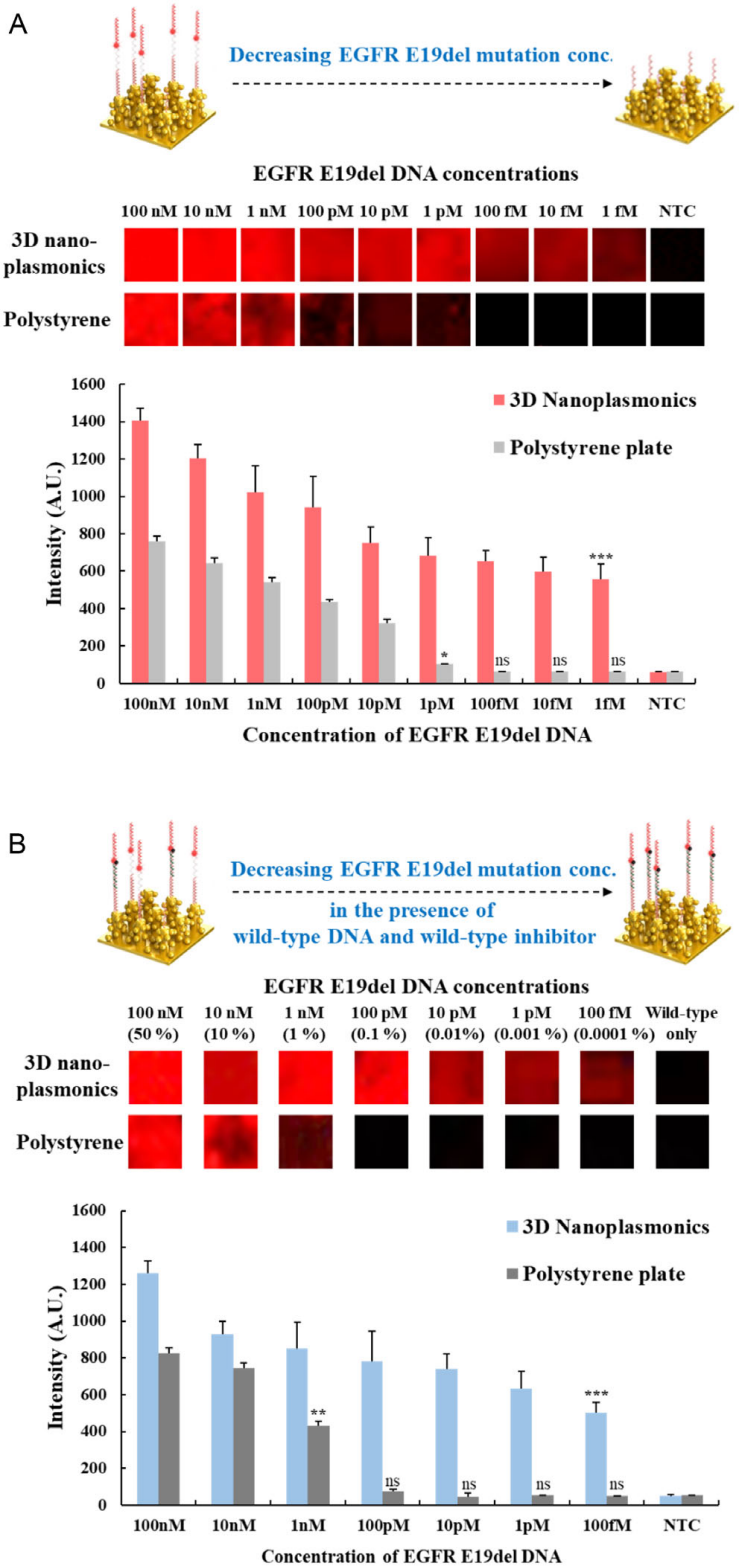
Plasmon‐enhanced fluorescence (PEF) effect of the 3D‐nanoplasmonic substrate for DNA detection. Fluorescence signals of the Cy5–probe hybridized to mutant DNA on the nanoplasmonic substrate in comparison with those of the polystyrene plate in A) the absence or B) presence of wild‐type DNA and inhibitor.

Plasmonic enhancement is a phenomenon in which the interaction of light with metallic nanostructures intensifies the electromagnetic fields at an optimal distance of 5–90 nm.^[^ ^22^ ^]^ The PEF effect generally known to decrease as the distance between the fluorescent molecule and the plasmonic surface increases.^[^ ^23^ ^]^ Figure S2, Supporting Information, compares PEF effects at different distances (50, 80, 100, and 150 bp) from the 3D‐nanoplasmonic substrate, using the same Cy5‐labeled DNA concentration, resulting in a reduction in PEF efficacy with increasing distance from the substrate. To design probes for comprehensive ctDNA detection and effective PEF, the Cy5 label was designed to face the nanoplasmonic substrate and positioned ≈25 nm away from the surface (5 nm for streptavidin and ≈20 nm for 56 bp DNA), as shown in Table 1 and Figure 1. The Cy5‐labeled probe and DNA template for the EGFR E19del mutation were hybridized on a nanoplasmonic substrate‐pre‐immobilized capture probe. This configuration resulted in distinct fluorescence signals on the nanoplasmonic substrate that were detectable at concentrations as low as 1 fM, even without nucleic acid amplification (**Figure** **2**A). We evaluated the PEF effect of the nanoplasmonic substrate on DNA detection by comparing the detection limit with that of a polystyrene plate, which is commonly used in enzyme‐linked immunosorbent assay. Polystyrene plate exhibited a detectable fluorescence signal down to 1 pM under the same conditions. Thus, the nanoplasmonic substrate demonstrated an ≈1000‐fold enhancement in DNA detection sensitivity through the PEF effect.

We further assessed the detection limit for EGFR E19del mutant DNA in the presence of EGFR E19 wild‐type DNA (100 nM) and excess of quencher‐labeled wild‐type inhibitor (10 μM). As shown in Figure 2B, on the nanoplasmonic substrate, the EGFR E19del mutant DNA was detectable down to 100 fM (0.0001% of EGFR E19del mutant DNA relative to wild‐type DNA), indicating a 100‐fold decrease in sensitivity compared to that in the absence of EGFR E19 wild‐type DNA. In the presence of wild‐type DNA (100 nM) alone, no fluorescent signal was observed due to the presence of excess wild‐type inhibitor quencher. Therefore, the fluorescence signal was confirmed to originate from the hybridization of the EGFR E19del DNA.

In contrast, on the polystyrene plate, the EGFR E19del mutant DNA was detectable only down to 1 nM (1% of EGFR E19del mutant DNA relative to wild‐type DNA), indicating a 1000‐fold decrease in sensitivity compared to that in the absence of EGFR E19 wild‐type DNA. When wild‐type DNA was present, the detection limit of EGFR E19del mutant DNA on the nanoplasmonic substrate increased less than that on the polystyrene plate. This phenomenon might not solely be attributed to the PEF effect of the nanoplasmonic substrate but also to the increased volume‐to‐surface ratio resulting from its 3D structure. This structural aspect facilitated a greater number of binding sites for the capture and fluorescent probes. Consequently, we confirmed that the nanoplasmonic substrate led to a 10^4^‐fold improvement in mutation detection sensitivity compared to the polystyrene substrate in the presence of the wild type.

### 2.3 Amplification‐Inhibiting Effect of Wild‐Type Inhibitor

Next, we evaluated the amplification inhibition effect of the wild‐type inhibitor on wild‐type DNA compared to mutation DNA. SYBR Green dye‐based real‐time PCR was conducted for EGFR E19del DNA (ranging from 100 to 100 zM) and its wild‐type DNA (100 and 10 fM) using the same primers listed in Table 1, in the presence of excess wild‐type inhibitor (10 μM). As shown in Figure S3A, Supporting Information, EGFR E19del DNA was well amplified with a Ct value of 24.1, even at 100 zM, exhibiting a linear decrease in Ct values with increasing EGFR E19del DNA concentrations. In contrast, although the wild‐type DNA was amplified, a significant increase in Ct values was observed. At a concentration of 100 fM, the Ct value of wild‐type DNA was 17.5, indicating a 14.03 increase compared with the 3.47 Ct value of EGFR E19del DNA. Similarly, at 10 fM, the Ct value of wild‐type DNA was 23.56, showing a 15.5 increase compared to the 8.06 Ct value of EGFR E19del DNA. To achieve the same Ct value, the concentration of wild‐type DNA was ≈10^4^ to 10^5^ times higher than that of EGFR E19del DNA. (Figure S3B, Supporting Information). These results confirmed that the wild‐type inhibitor significantly hindered the amplification of wild‐type DNA, which can enhance the detection sensitivity of mutant DNA in the presence of wild‐type DNA. Gel electrophoresis further validated proper amplification, revealing a size difference between the wild‐type amplicon (100 bp) and EGFR E19del amplicon (88 bp) as shown in Figure S3C, Supporting Information.

### 2.4 Analytical Sensitivity for EGFR Mutation Detection

**Figure 3:**
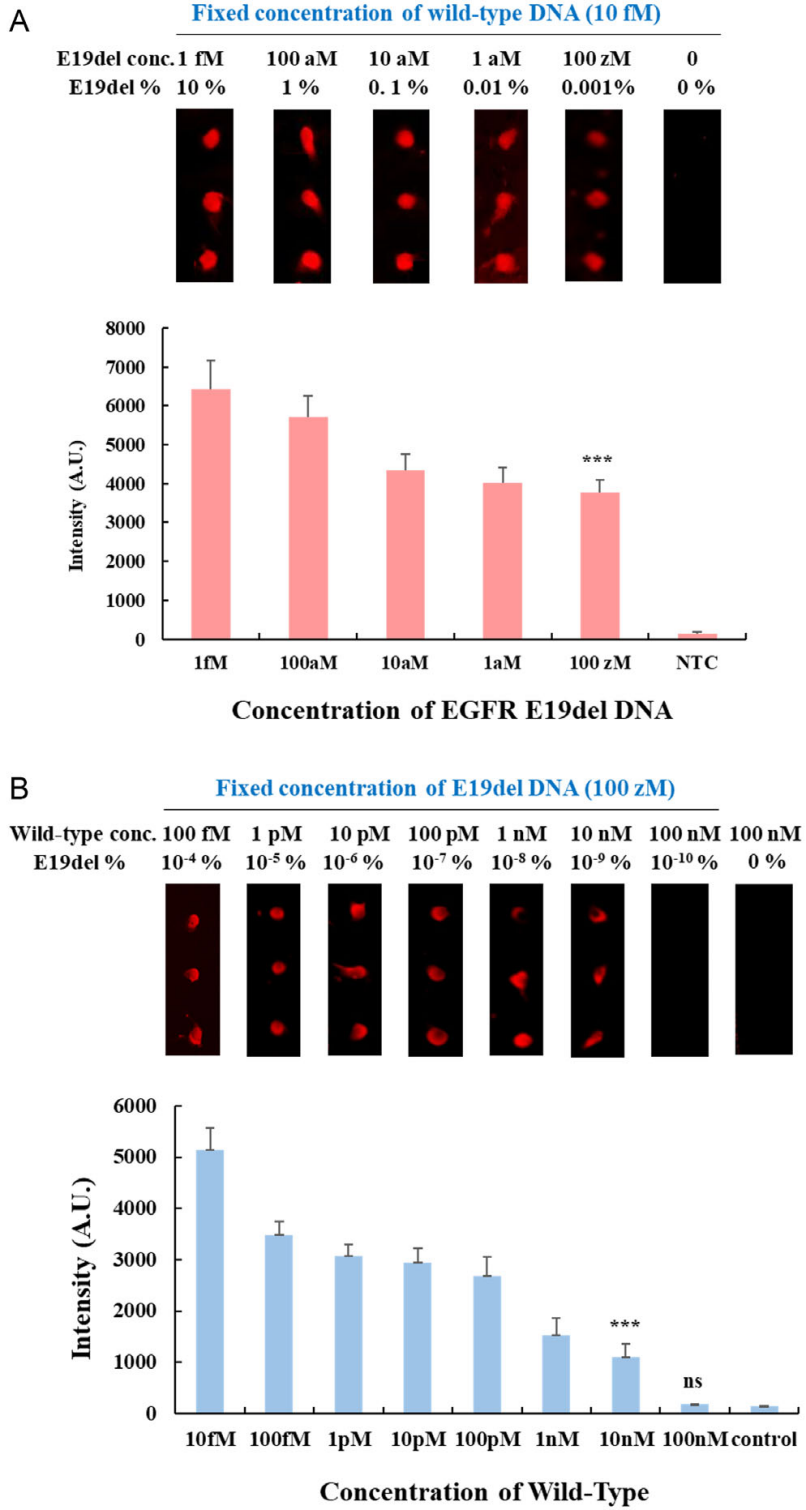
Analytical sensitivity test for EGFR exon 19 deletion (E19del) mutation detection. Fluorescence signals after recombinase polymerase amplification (RPA) hybridized on the nanoplasmonic substrate according to A) EGFR E19del DNA concentration at a fixed concentration of the wild‐type DNA (10 fM) and B) wild‐type concentration at fixed concentration of the EGFR E19del DNA (100 zM).

We evaluated the analytical sensitivity of detecting EGFR mutant DNA in the presence of wild‐type DNA after nucleic acid amplification and hybridization on the 3D‐nanoplasmonic substrate. First, PCR (for 40 cycles) and RPA (for 30 min) were performed for EGFR E19del DNA ranging from 1 fM to 100 zM in the presence of wild‐type DNA at a fixed concentration of 10 fM and the quencher‐labeled wild‐type inhibitor at a fixed concentration of 10 μM. Since each primer was used at 500 nM, the total amplicon concentration generated from mutant DNA and wild‐type DNA was capped at 500 nM. The amplicons were treated with lambda exonuclease and mixed with a fluorescent probe and an additional wild‐type inhibitor to achieve complete fluorescence quenching of the wild‐type DNA. Finally, the mixture is hybridized onto the capture‐probe‐immobilized nanoplasmonic substrate. **Figure** **3**A and S4, Supporting Information, show clear fluorescence signals of the EGFR E19del amplicon from PCR and RPA, respectively, even at a low concentration of 100 zM (3 copies/reactions, 0.001%). The fluorescence signals decreased slightly as the concentration of EGFR E19del DNA decreased because of the increased proportion of amplicons from wild‐type DNA. When only the wild‐type was used as a control, there was no distinguishable fluorescence signal due to quenching by the wild‐type inhibitor. Therefore, we confirmed that the fluorescence signals originated solely from E19del DNA amplicons.

Furthermore, to determine the percentage detection limit of the mutant DNA compared to wild‐type DNA, we fixed the concentration of EGFR E19del DNA at 100 zM (3 copies/reactions) and increased the concentration of wild‐type DNA from 100 fM to 100 nM. As shown in Figure 3B, distinct fluorescent signals from the EGFR E19del were detectable in the presence of wild‐type DNA up to 10 nM, indicating a mutant DNA frequency of 1 × 10^−9^% (100 zM mutation/10 nM wild‐type). In contrast, no fluorescent signal was detected for the EGFR E19del in the presence of wild‐type DNA at 100 nM or when only wild‐type DNA was present at 100 nM.

We also evaluated the analytical sensitivity for detecting EGFR E20ins and E21 L858R mutant DNAs. As shown in Figure S5 and S6A, Supporting Information, similar to EGFR, E19del, E20ins, and E21 L858R demonstrated detectability down to 100 zM (3 copies/reactions, 0.001%) in the presence of wild‐type DNA at a fixed concentration of 10 fM. Furthermore, E21 L858R DNA at 100 zM (3 copies/reactions) also showed detectability in the presence of wild‐type DNA up to 10 nM, implying a mutation detection sensitivity of 1 × 10^−9^% compared to wild‐type (Figure S6B, Supporting Information). We did not evaluate the detection limit for E20ins further because it had a hybridization structure similar to that of E19del. The Cy5 label positions for EGFR E19del and E20ins varied depending on the mutation; however, it was anticipated that the distance from the surface would not differ significantly, resulting in comparable sensitivity.

The mutation frequency sensitivity was compared with that of the real‐time PCR technique for EGFR E21 L858R DNA. The comparison was conducted using EGFR E21 L858R DNA template concentrations ranging from 1 fM to 100 zM in the presence of wild‐type DNA at a fixed concentration of 10 fM using the same primers listed in Table 1, without a wild‐type inhibitor. As shown in Figure S7, Supporting Information, the wild‐type DNA showed nonspecific amplification with a Ct value of 33.78. As the concentration of E21 L858R DNA decreased, the Ct value increased, and at 100 zM (0.001%), it was difficult to be distinguished from wild‐type DNA. Therefore, the mutation frequency sensitivity of real‐time PCR was ≈0.01% compared to the wild type, consistent with values previously reported.^[^ ^12^ ^]^ Furthermore, a recovery study was performed using normal plasma samples (200 μL) spiked with EGFR E21 L858R DNA template at concentrations ranging from 100 fM to 100 zM. cfDNA was extracted from the plasma samples using a commercialized kit (MagListo cfDNA Extraction Kit), and the cfDNA was analyzed by our assay method and real‐time PCR. As shown in Figure S8, Supporting Information, the 3D plasmonic substrate exhibited distinguishable fluorescence signals from 10 fM of EGFR E21 L858R DNA in plasma, while real‐time PCR did not amplify in any of the spiked EGFR E21 L858R DNA samples nor in the non‐spiked normal plasma sample. From these results, we confirmed the significantly higher mutation frequency sensitivity of the 3D‐nanoplasmonic‐based assay method.

### 2.5 Analytical Specificity of EGFR Mutation Multiplex Assay Chip

**Figure 4:**
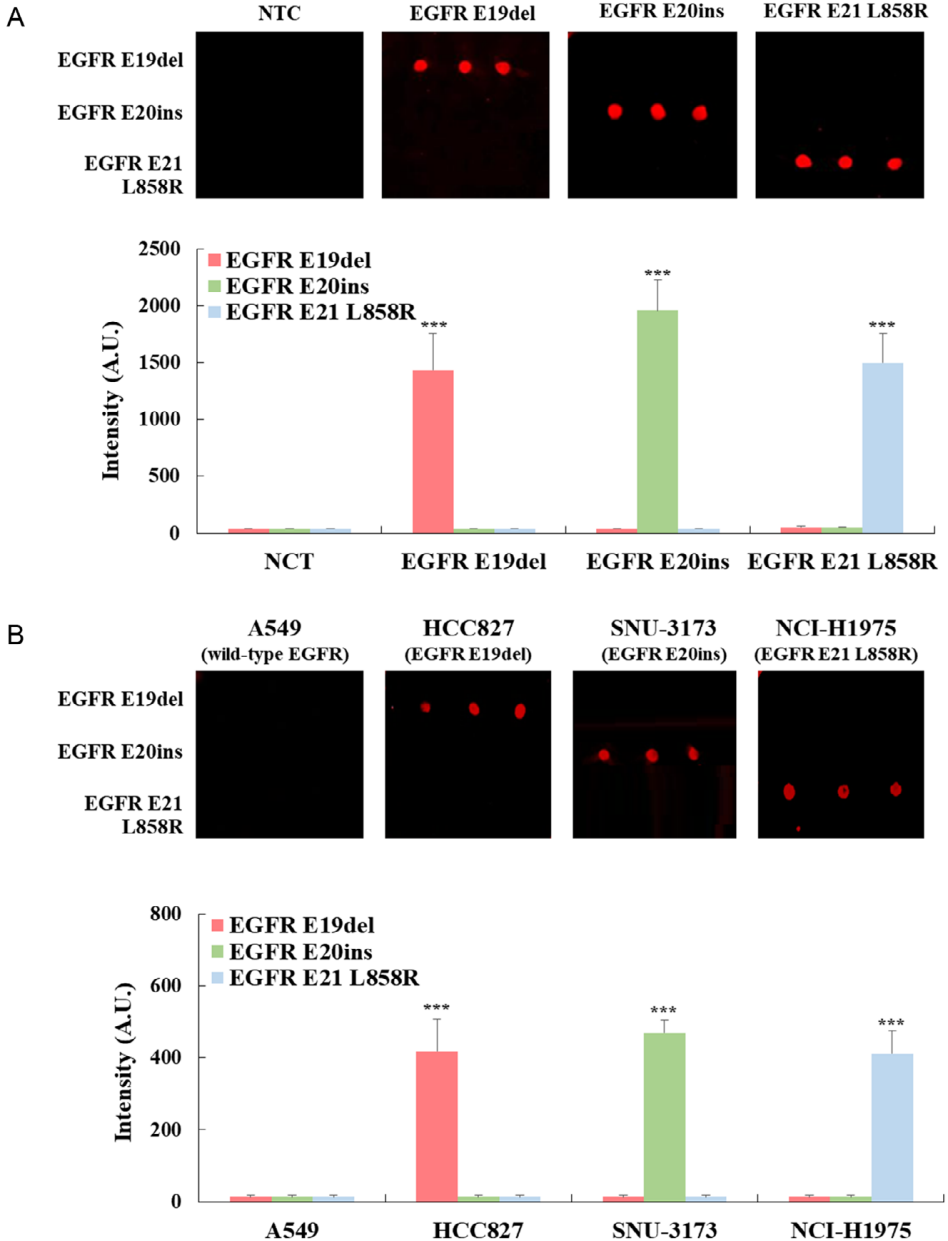
Analytical specificity tests using A) synthetic templates and B) DNA extracted from cancer cells for the 3D‐nanoplasmonic‐based EGFR mutation multiplex assay chip.

For analytical specificity testing, an EGFR mutation multiplex assay chip was fabricated. A 3D‐nanoplasmonic microarray was prepared by immobilizing the capture probes on the nanoplasmonic substrate (8 × 8 mm) in a 3 × 3 array with a spotting volume of 50 nL. The 3D‐nanoplasmonic microarray was affixed to a glass slide, and a PDMS chamber was fabricated to contain the solution (Figure S9, Supporting Information). And, three‐multiplex RPA was performed using three primer sets (each at 500 nM) and three wild‐type inhibitors (each at 10 μM) for the detection of E19del, E20ins, or E21 L858R. Each target template (E19del, E20ins, or E21 L858R) was added at a concentration of 10 fM. Following lambda exonuclease treatment and hybridization on the EGFR mutation multiplex assay chip, fluorescence signals were detected exclusively at the spots where the target capture probes were immobilized for E19, E20, and E21, as shown in **Figure** **4**A. Conversely, in the absence of the target template, no distinguishable fluorescence signals were observed at any spot. We evaluated the analytical specificity using extracted DNA from lung cancer cell lines: HCC827 harboring the E19del mutation (E746‐A750del),^[^ ^24^ ^]^ SNU‐3173 harboring the E20ins mutation (H773insAH),^[^ ^25^ ^]^ NCI‐H1975 harboring the E21 L858R mutation,^[^ ^26^ ^]^ and A549 with wild‐type EGFR.^[^ ^27^ ^]^ As shown in Figure 4B, HCC827, SNU‐3173, and NCI‐H1975 exhibited fluorescence signals at the spots corresponding to E19del, E21ins, and E21 L858R, respectively, while A549 showed no distinguishable fluorescence signals at any spot. Therefore, we confirmed the high specificity of the 3D‐nanoplasmonic‐based EGFR mutation multiplex assay chip for E19del, E20ins, and E21 L858R. Therefore, we confirmed the high specificity of the 3D‐nanoplasmonic‐based EGFR mutation multiplex assay chip for E19del, E20ins, and E21 L858R.

### 2.6 Clinical Sample Test Using Plasma Samples

The clinical effectiveness of the 3D‐nanoplasmonic‐based EGFR mutation multiplex assay chip was evaluated using 52 plasma samples from patients with malignant lung tumor (*n* = 21) and benign lung tumor (*n* = 10) and normal individuals (*n* = 21). The clinical characteristics of the patients are shown in Table S1, Supporting Information. The histological type and cancer stage were confirmed using clinical tissue biopsy based on histological staining. Malignant lung tumors were classified into stage 1 (*n* = 3), stage 2 (*n* = 4), stage 3 (*n* = 7), and stage 4 (*n* = 7).

For sensitivity comparison, we analyzed cfDNAs extracted from the plasma samples of patients with malignant lung tumors by NGS using ion‐torrent‐based whole‐exome sequencing for a 53‐gene panel including EGFR, as listed in Table S2, Supporting Information. Table S3, Supporting Information, presents the full NGS results, including mutated genes, their DNA sequences, corresponding protein regions, variant allele frequency (VAF) (%), and sequencing depths (*X*). The NGS analysis detected mutations in only 8 out of 21 malignant lung tumor patients, resulting in a clinical sensitivity of 38% (8/21) compared to the tissue biopsy results. The mutation detected patients showed VAFs ranging from a minimum of 0.5% to a maximum of 53%. As summarized in **Table** **2**, when examining the clinical sensitivity at each stage, NGS did not detect any patients in stage 1, detected only one patient in stage 2 (patient #7), resulting in clinical sensitivities of 0% (0/4) and 25% (1/4), respectively. The remaining seven patients belonged to late stages: two patients (patient #8 and #13) with stage 3 and five patients (patients #17–21) with stage 4, resulting in clinical sensitivity of 28.6% (2/7) and 71.4% (5/7), respectively. The quantity of ctDNA has been known to vary depending on the stage of cancer progression, with levels expected to be relatively low (<0.01%) in early stages but further increasing in advanced stages due to increased shedding and destruction of cancer cells, reflecting tumor burden.^[^ ^28^ ^]^ Therefore, The limited detection of mutations in the majority of early‐stage cancer patients by NGS analysis might be attributed to its low sensitivity.

**Table 2:** Comparison of the clinical sensitivity and specificity of our assay and next‐generation sequencing (NGS).

Sample Methods			Malignant lung tumor (*n* = 21)				Benign lung tumor (*n* = 10) + Normal individuals (*n* = 21)
			Stage 1 (*n* = 3)	Stage 2 (*n* = 4)	Stage 3 (*n* = 7)	Stage 4 (*n* = 7)	
Our assay		Detected number	3	4	7	7	0
		Sensitivity at each stage	100%	100%	100%	100%	Specificity 100%
		Total sensitivity	100%				–
NGS	53‐pannel	Detected number	0	1	2	5	
		Sensitivity at each stage	0%	25%	28.6%	71.4%	
		Total sensitivity	38%				
	EGFR	Detected number	0	0	1	4	
		Sensitivity at each stage	0%	0%	14.3%	57%	
		Total sensitivity	24%				

Among the eight patients who had mutations in the NGS analysis, EGFR mutations were observed in five patients (patients #8, 17, 19–21), resulting in a clinical sensitivity of 24% (5/21), and clinical sensitivity for stage 1 of 0% (0/3), stage 2 of 0% (0/4), stage 3 of 14.3% (1/7), and stage 4 of 57% (4/7). Specifically, two patients (patients #8 and #17) harbored the E19del mutation (c.2236_2250del and c.2235_2249del), whereas three patients (patients #19–21) harbored the E21 L858R mutation.

**Figure 5:**
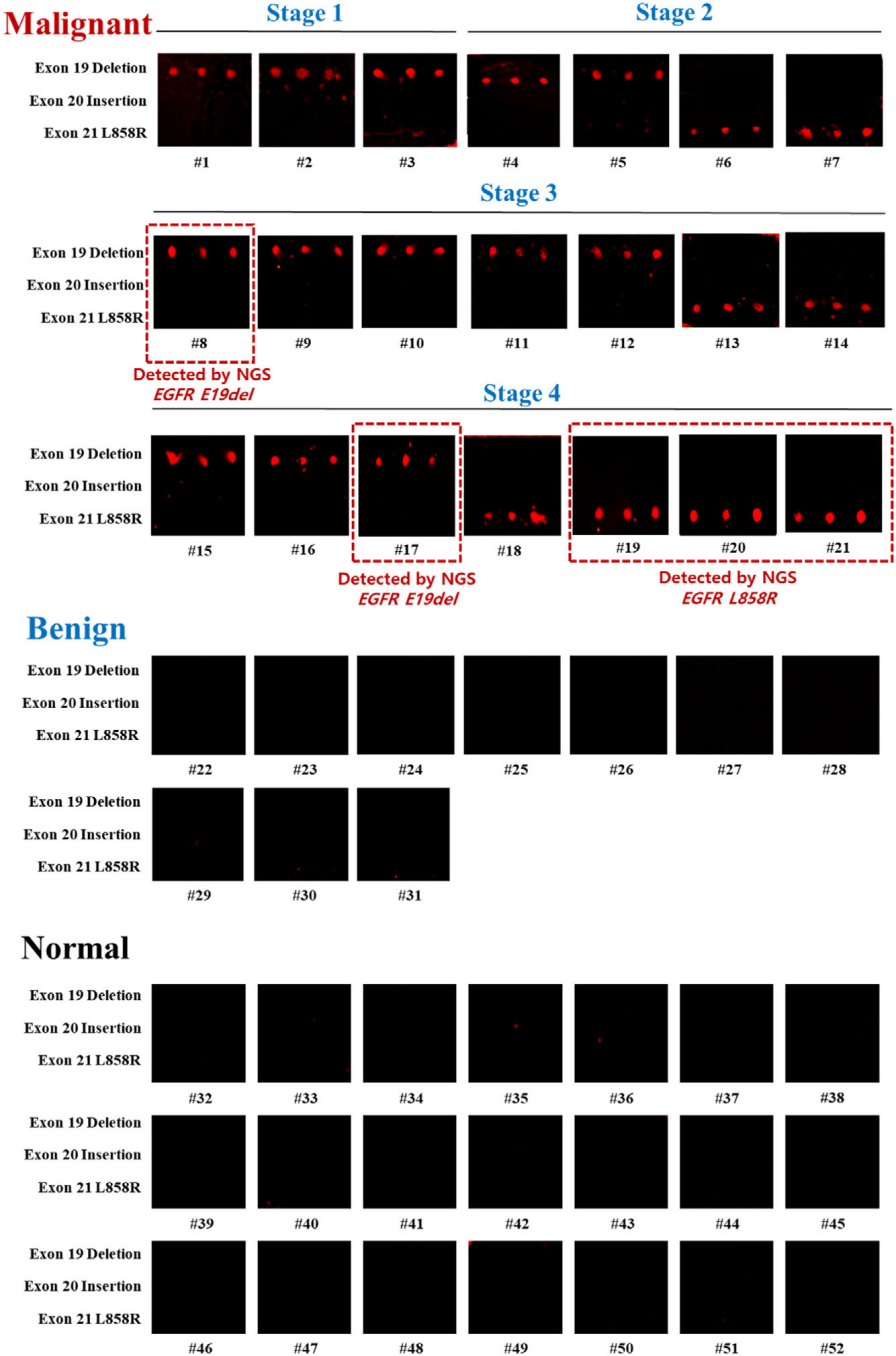
Clinical effectiveness test using plasma samples from patients with malignant lung tumor and benign lung tumor, and normal individuals.

We assessed the 3D‐nanoplasmonic‐based EGFR mutation multiplex assay chip using the cfDNAs extracted from plasma samples. Cell‐free DNAs were extracted using the commercial cfDNA extraction kit, which have concentrations ranging from 10 to 50 ng μL^−1^. We performed 3‐plex RPA using the cfDNA, lambda exonuclease treatment, and hybridization on the 3D‐nanoplasmonic microarray. **Figure** **5** shows that the EGFR mutation multiplex assay chip showed strong fluorescent signals across all patients with malignant lung tumor, resulting in a clinical sensitivity of 100% (21/21). Among the patients with malignant lung tumor, 62% patients showed EGFR E19del (13/21) and 38% patients showed EGFR E21 L858R mutation (8/21). All 21 patients with malignancies had only one type of EGFR mutation. The tested samples did not show E20ins (0/21). In contrast, no detectable fluorescence signals were observed in all patients with benign lung tumor and normal individuals of tumor free, resulting in a clinical specificity rate of 100% (31/31). Therefore, the results showed 100% accuracy with tissue biopsy results. Moreover, the types of EGFR mutations detected by NGS matched precisely with those identified using the EGFR mutation multiplex assay chip. Despite testing a small number of clinical samples, our EGFR mutation multiplex assay chip successfully differentiated all patients with malignant lung tumors, including those in early stages 1 and 2.

This was attributed to its extremely high analytical sensitivity and the detection capability of all deletions and insertions within the target region. Based on our findings, it is anticipated that EGFR mutations will be detected in a significantly larger proportion of lung cancer patients than previously reported in the literature.

Figure S10, Supporting Information, shows a summary plot of fluorescence intensity for malignant samples, comparing different mutation types and cancer stages. In the analysis, the EGFR E21 L858R mutation demonstrated an increasing trend in signal intensity corresponding to cancer stage, while EGFR E19del exhibited no correlation with cancer stage. Since our strategy involves utilizing a wild‐type inhibitor that can impede amplification and suppress the fluorescence of the wild type, highly sensitive diagnosis of malignant lung cancer patients was possible. However, precise quantification of ctDNA compared to the wild type might be difficult.

We compared our assay with other mutation assays as shown in **Table** **3**. Although direct sequencing and NGS detect various mutations, their sensitivity is notably low, typically in the range of 1%–10%.^[^ ^11^, ^29^ ^]^ Digital PCR, a recently developed method with enhanced sensitivity (0.01%), requires numerous primers for the detection of various mutations.^[^ ^11^, ^30^ ^]^ In contrast, our assay method showed remarkable sensitivity of 1 × 10^−9^%, surpassing that of digital PCR by 10^7^‐fold. According to our investigation, this sensitivity represents the highest level reported. Utilizing our exceptionally sensitive technology, early diagnosis is feasible.

**Table 3:** Comparison of the analytical sensitivity of our assay with that of other mutation assay methods.

Methods	Mutation frequency sensitivity [%]	Number of detectable deletions or insertions using one primer–probe set	References
Our assay	1 × 10^−9^	All mutations in the target region (In this study, all deletions occurred within 26 bp and all insertions occurred within 45 bp)	–
Digital PCR	0.01	1	[11, 23]
Peptide nucleic acid–locked nucleic acid (PNA–LNA) PCR clamp	0.01	1	[31]
PCR invader	0.1	1	[32]
CastPCR	0.1	1	[33]
Real‐time PCR	0.1	1	[34]
NGS	1	All mutations	[11]
Direct sequencing	10	All mutations	[29]

## 3 Conclusion

In summary, we developed a 3D‐nanoplasmonic‐based EGFR mutation multiplex assay chip for detecting EGFR E19del, E20ins, and E21L858R point mutations with exceptional sensitivity. Compared to previously reported EGFR detection methods with a sensitivity range of 0.01%–1%, our approach achieved a superior higher sensitivity of 1 × 10^−9^% mutant frequency due to the synergistic effects of the PEF of the 3D‐nanoplasmonic and wild‐type inhibitor. Furthermore, our method facilitated the detection of all deletions or insertions within the target region using a single primer and probe set, leading to the identification of numerous mutations in various individuals with enhanced clinical sensitivity through a cost‐effective assay. Based on this synergistic effect, clinical plasma ctDNA testing using the 3D‐nanoplasmonic‐based EGFR mutation multiplex assay chip not only diagnosed malignant tumors from stages 1 to 4, but also accurately distinguished benign and normal cases from malignant cases. As a result, it achieved 100% sensitivity and specificity. Furthermore, our method takes ≈70 min post‐DNA extraction, and the total process takes around 2 h, including cfDNA extraction. This timeframe is shorter than that of the real‐time PCR‐based approach (cobas EGFR Mutation Test), which typically takes around 4 h. Therefore, our economical and effective rapid analysis method with high accuracy can aid in early cancer diagnostic screening, elimination of unnecessary tissue examinations, and monitoring of the therapeutic efficacy of EGFR‐targeted treatments and cancer recurrence in clinical settings. Furthermore, our 3D‐nanoplasmonic‐based mutation multiplex assay chip, which functions as a microarray with the ability to immobilize various capture probes, can be readily applied for the detection of various cancer biomarkers.

## 4 Experimental Section

4.1

4.1.1

##### Materials

Polyethylene terephthalate (PET) film with a thickness of 188 μm was purchased from TORAY INDUSTRIES, Inc. (Tokyo, Japan). A 97% solution of 1H, 1H, 2H, 2H‐perfluorodecanethiol (PFDT) was purchased from Sigma Aldrich (St. Louis, MO, USA). Au was obtained from iTASCO (Seoul, Republic of Korea). Synthetic DNA templates, primers, and probes and MagListo cfDNA Extraction Kit were purchased from Bioneer (Daejeon, South Korea). The TwistAmp Basic Kit for RPA was purchased from TwistDx (Babraham, UK). PCR Master Mix (2X) and lambda exonuclease were purchased from Thermo Fisher (Carlsbad, CA, USA).

##### Fabrication of the 3D‐Nanoplasmonic Substrate

PET film was subjected to Ar plasma treatment for 2 min using a customized radio frequency (RF) ion‐etching instrument (LAT, Korea) with the following fixed parameters: 5 sccm Ar flow, 80 mTorr pressure, and a plasma power of 100 W. A 100 nm thick Au layer was then deposited on the PET nanopillars at a rate of 2.0 Å s^−1^ under a base pressure of 9.6 × 10^−6^ Torr using a thermal evaporation system (LAT, Korea). Subsequently, PFDT was applied, followed by the thermal evaporation of Au onto the PFDT‐treated Au/PET nanopillar substrate at 0.3 Å s^−1^. The 3D plasmonic substrate was cut into 8 × 8 mm pieces to fabricate the assay chip.

##### Preparation of the EGFR Mutation Multiplex Assay Chip

A mixture containing 1 μL of streptavidin (0.1 mg mL^−1^), 5 μL of each biotin‐modified oligonucleotide capture probe (10 μM) targeting the exons 19, 20, and 21, and 14 μL of diethylpyrocarbonate (DEPC)‐treated water was precisely spotted onto the 3D‐nanoplasmonic substrate (8 × 8 mm) at a volume of 50 nL using BIOSPOT Custom (BioFluidix, Germany). Nine (3 × 3) subarrays were arranged with a spacing of 1.5 mm between the individual spots. The nanoplasmonic chip was incubated overnight in a refrigerator at 4 °C for immobilization and washed thrice with DEPC‐treated water to remove the unbound streptavidin and capture probes, followed by incubation with 0.5% BSA solution at 25 °C for 2 h to cover the non‐spotted areas. Then, the chip was washed thrice with DEPC‐treated water and stored in a refrigerator at 4 °C until use.

##### Nucleic Acid Amplification

For PCR, PCR Master Mix (2X), 500 nM of each forward primer, and a 5′‐end phosphorylated reverse primer, along with 10 μM of each 3′‐end quencher (Black Hole Quencher (BHQ) 2)‐labeled wild‐type inhibitor, were mixed with the mutated and wild‐type DNA templates, resulting in a total volume of 20 μL. PCR was performed using the CFX96 real‐time system (BioRad, CA, USA) under the following conditions: 95 °C for 5 min, followed by 40 cycles of 95 °C for 1 min 30 s, 59 °C for 30 s, and 72 °C for 60 s. The total cycling time was ≈2 h and 30 min.

RPA reaction was conducted using TwistAmp Liquid Basic, following the manufacturer's protocol with minor adjustments. The concentrations of the primers and probes were the same as those used for PCR. Similarly, the total volume was adjusted to 20 μL. The reactions were carried out at a constant temperature of 39 °C for 30 min using ThermoMixer C (Eppendorf, Hamburg, Germany).

##### Hybridization onto the EGFR DNA Mutation Multiplex Assay Chip

After amplification, 1 μL of the RPA solution was combined with 1 μL of lambda exonuclease, 5 μL of 10× reaction buffer, and DEPC‐treated water, resulting in a reaction volume of 50 μL. Then, the solution was incubated for 30 min at 37 °C. Subsequently, 1 μL of each fluorescent probe (5 μM) and 1 μL of each wild‐type inhibitor (500 μM) were added to the resulting solution, and the mixture was applied to the EGFR multiplex assay chip in a sealed container and incubated for 10 min at 37 °C for hybridization. Finally, the chip was washed thrice with DEPC water and dried.

##### Fluorescence Detection

Hybridized chip on slide glass was scanned using the InnoScan 710 microarray scanner at a laser excitation wavelength of 635 nm (Innopsys, Carbonne, France). The negative control was always contained on the same slide and scanned at the same time. Data intensities were extracted using the Mapix software, and data analysis was performed for each chip at condition of brightness 21, contrast 77, and balance 0. In the case of microarray analysis, we confirmed the absence of fluorescent signals from the spot areas against the background by maximizing the brightness to 100.

##### Clinical Sample Test

Blood samples were collected from patients (*n* = 31) suspected of having lung cancer by computed tomography, positron emission tomography, or bronchoscopy before treatment at the Samsung Medical Center, with the explicit consent of the participants for their involvement in the research (IRB approval number: SMC 2021‐06‐083‐015). Blood samples were collected using ethylenediaminetetraacetic‐acid‐treated vacuum tubes and immediately stored at 4 °C. Within 8 h of collection, plasma was separated via centrifugation (1600 g, 10 min, 4 °C), transferred to sterilized tubes, and stored at –80 °C until analysis. The pathological classification was confirmed via surgical‐biopsy‐based histological staining techniques of tissue slides. We obtained plasma samples from 21 normal individuals without tumors from Innovative Research (FL, USA).

cfDNA was isolated from the plasma samples using the commercial MagListo cfDNA Extraction Kit. For RPA amplification, 1–2 μL of each cfDNA was used as per the protocol described earlier. For comparison, NGS panel analysis targeting 53 plasma cfDNA genes was performed using the Ion Torrent technology.

##### Statistical Analysis

All fluorescence data (*n* = 3) were expressed as the mean ± SD. A one‐sided *t*‐test was employed to compare the difference in fluorescence signals between the limit of detection concentration and the negative control. Results were denoted as follows: “ns” indicated no statistical difference, “*” indicated statistical significance, with *P < 0.5, **P < 0.1, ***P < 0.01, and ****P < 0.001.

## Conflict of Interest

The authors declare no conflict of interest.

## Supporting information

Supplementary Material

## Data Availability

The data that support the findings of this study are available from the corresponding author upon reasonable request.
