# Supplementary material for: Highly Sensitive 3D‐Nanoplasmonic‐Based Epidermal Growth Factor Receptor Mutation Multiplex Assay Chip for Liquid Biopsy
Source: Small Sci. 2024 Jun 26;4(8):2400101. doi: 10.1002/smsc.202400101 (PMC11934998; doi:10.1002/smsc.202400101)

Supporting information

Highly Sensitive 3D Nanoplasmonic-Based EGFR Mutation Multiplex Assay Chip for Liquid Biopsy

Ji Young Lee,[1] Byeong-Ho Jeong,[2] Ho Sang Jung,[1] Taejoon Kang,[3,4] Yeonkyung Park,[3] Jin Kyung Rho,[5] Sung-Gyu Park,[1]* Min-Young Lee[1]*

[1]Advanced Bio and Healthcare Materials Research Division, Korea Institute of Materials Science (KIMS), 797, Changwon-daero, Seongsan-gu, Changwon-si, Gyeongsangnam-do, 51508, Republic of Korea

[2]Division of Pulmonary and Critical care medicine, Department of Medicine, Samsung Medical Center, SungkyunKwan University School of Medicine, 81, Irwon-ro, Gangnam-gu, Seoul, 06351, Republic of Korea

[3]Bionanotechnology Research Center, Korea Research Institute of Bioscience and Biotechnology (KRIBB), 125 Gwahak-ro, Yuseong-gu, Daejeon, 34141, Republic of Korea

[4]School of Pharmacy, Sungkyunkwan University, 2066, Seobu-ro, Jangan-gu, Suwon, Gyeonggi-do, 16419, Republic of Korea

[5]Department of Convergence Medicine, Asan Medical Center, University of Ulsan, College of Medicine, Olympic-ro 43-gil, Songpa-gu, Seoul, 05505, Republic of Korea

***Corresponding authors:**

Tel.: +82 55 280 3632, E-mail address: sgpark@kims.re.kr (S.G.Park)

Tel.: +82 55 280 3471, E-mail address: myay0615@ kims.re.kr (M.Y. Lee)

Figure S1. Scanning electron microscopy (SEM) images of the 3D nanoplasmonic substrate.

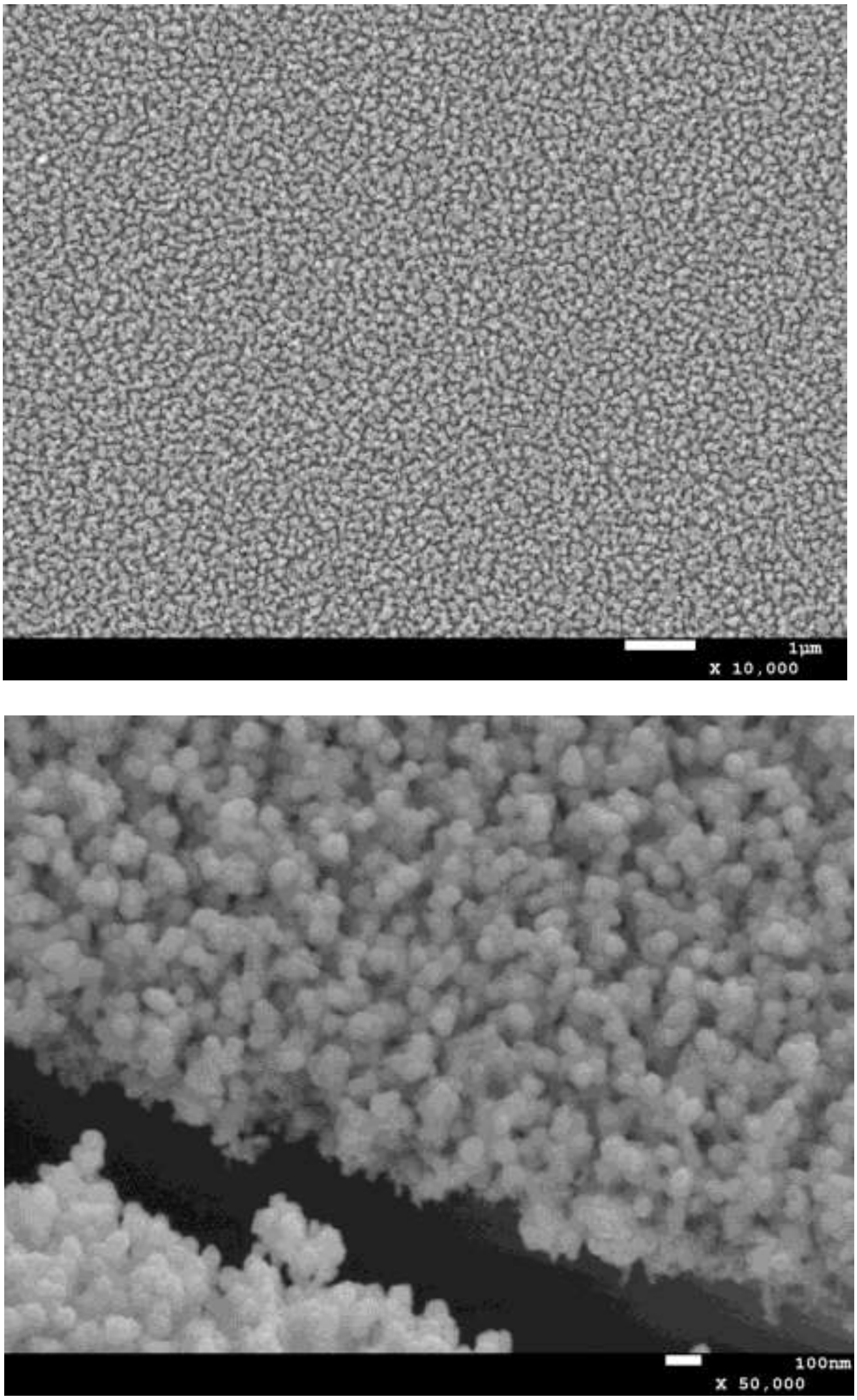

Figure S2. Normalized fluorescence of Cy5-labeled DNA on the 3D nanoplasmonic substrate according to DNA length.

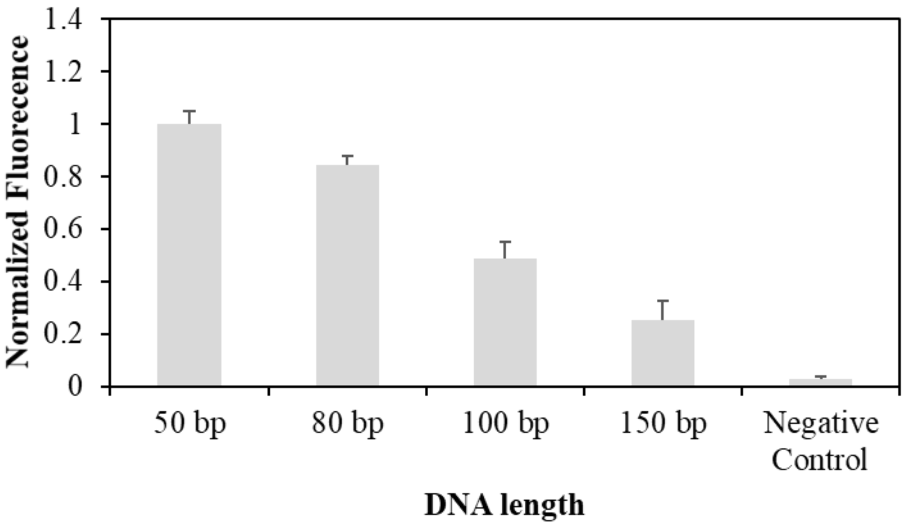

Figure S3. Amplification-inhibiting effect of the wild-type inhibitor. (A) Real-time polymerase chain reaction (PCR) amplification plot and Ct values for the mutated and wild-type DNA in the presence of the wild-type inhibitor. (B) Plot of Ct value vs. log template concentration. (C) Gel electrophoresis of the PCR products in the presence of the wild-type inhibitor (10 μM).

(A)

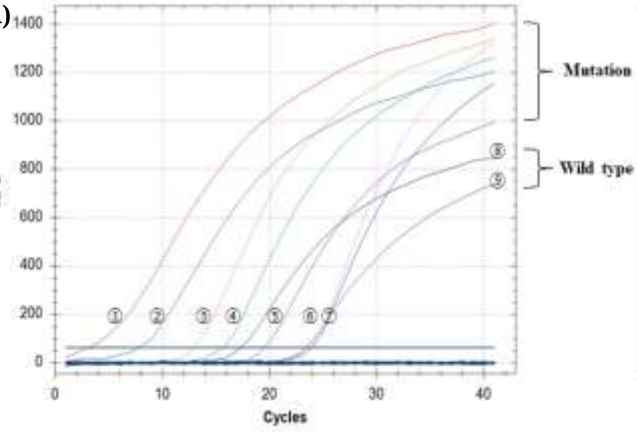

Wild-type inhibitor	Number	Template type	Concentration	Ct value
10 μM	①	Mutation (EGFR E19del)	100 fM	3.47
	②		10 fM	8.06
	③		1 fM	13.14
	④		100 aM	15.88
	⑤		10 aM	19.63
	⑥		1 aM	23.72
	⑦		100 zM	24.10
	⑧	Wild type (EGFR E19)	100 fM	17.50
	⑨		10 fM	23.56

(B) **(C)**

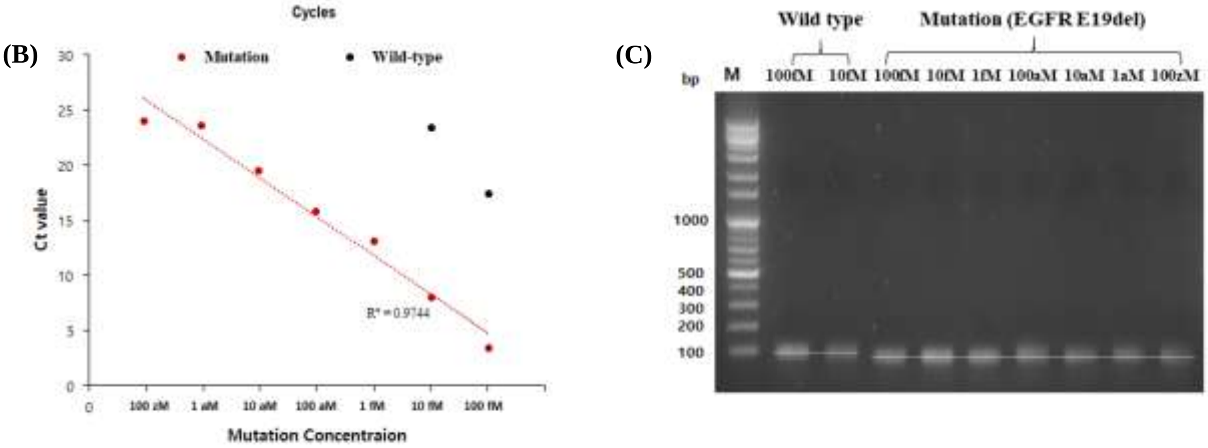

Figure S4. Analytical sensitivity test for epidermal growth factor receptor (EGFR) exon 19 deletion (E19del) mutation using PCR. Fluorescence signals after PCR hybridized on the nanoplasmonic substrate according to the EGFR E19del DNA concentration at a fixed concentration of the wild-type DNA (10 fM).

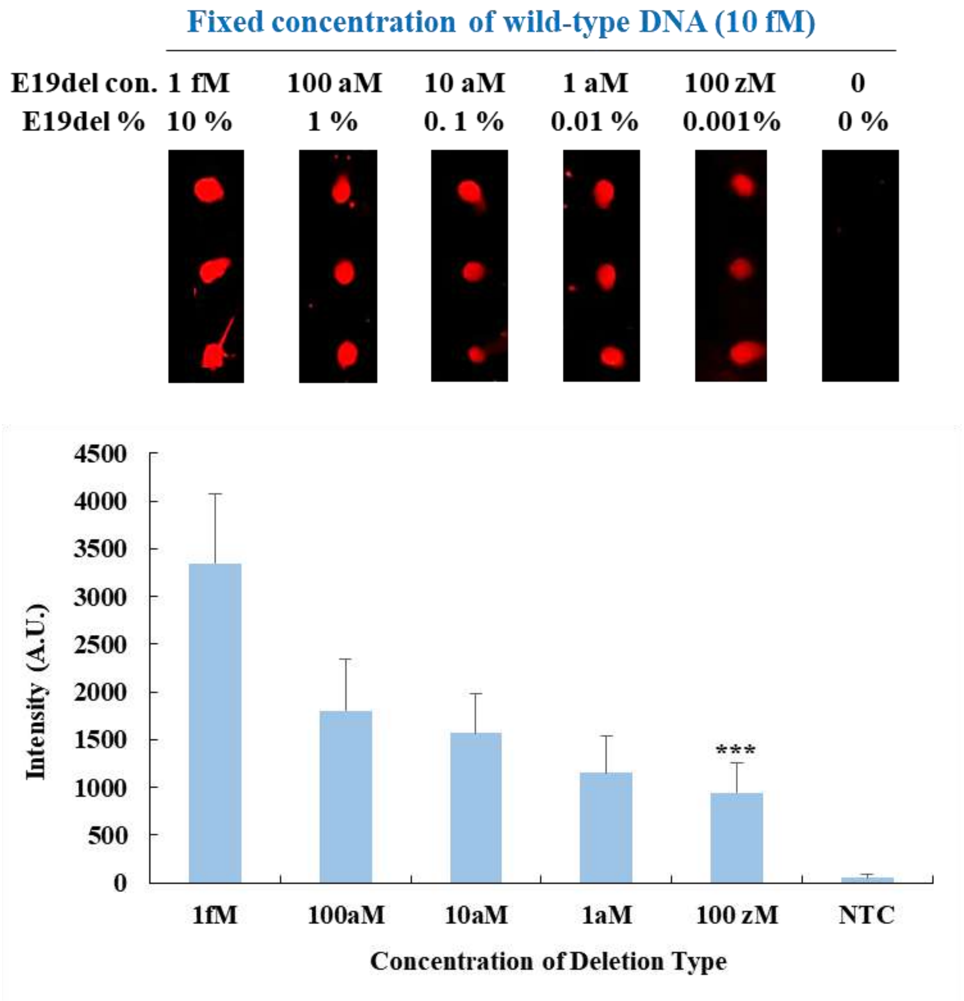

Figure S5. Analytical sensitivity test for EGFR exon 20 insertion (E20ins) mutation detection. Fluorescence signals after recombinase polymerase amplification (RPA) hybridized on the nanoplasmonic substrate according to the EGFR E20ins DNA concentration at a fixed concentration of the wild-type DNA (10 fM).

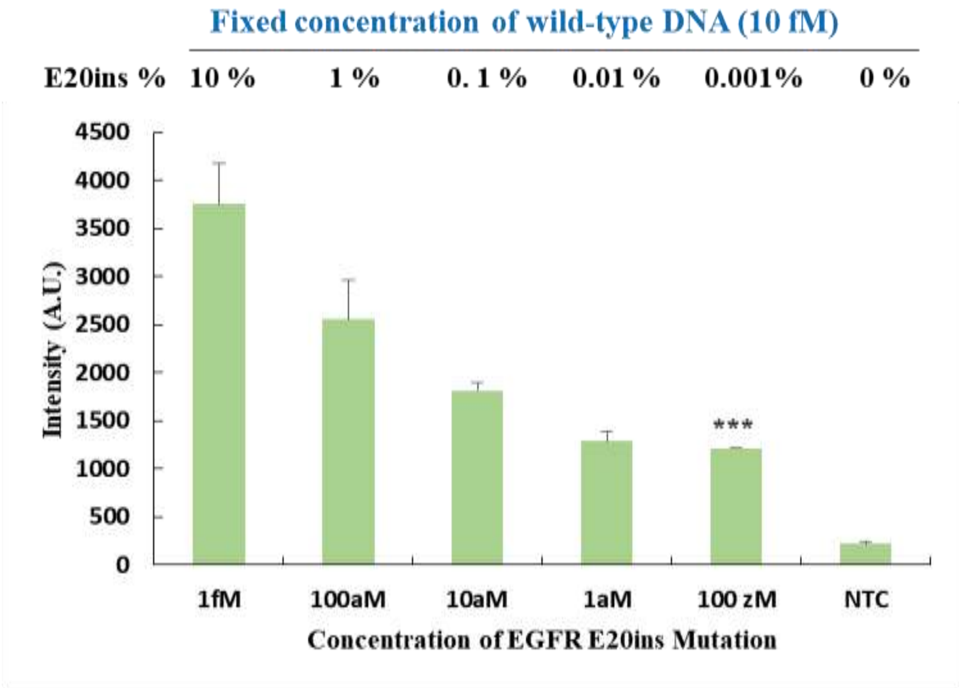

Figure S6. Analytical sensitivity test for EGFR exon 21 (E21) L858R mutation detection. Fluorescence signals after RPA hybridized on the nanoplasmonic substrate according to the **(A)** EGFR E21 L858R mutation concentration at a fixed concentration of the wild-type DNA (10 fM) and **(B)** wild-type concentration at a fixed concentration of the EGFR E21 L858R DNA (100 zM).

(A)

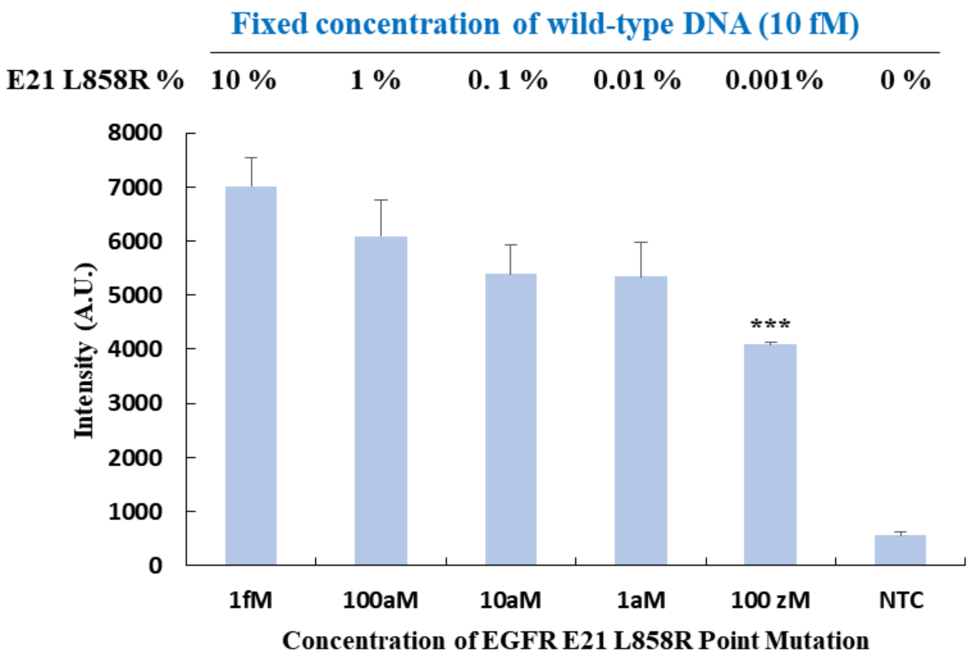

(B)

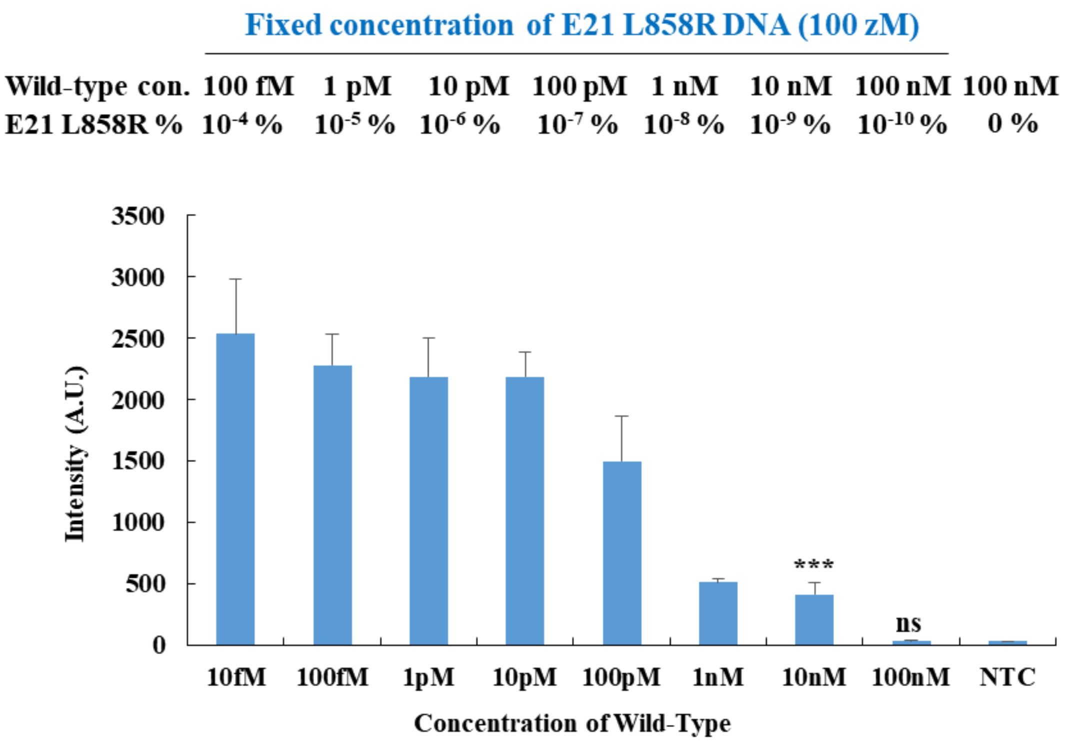

Figure S7. Real-time PCR analysis for E21 L858R mutation DNA in the presence of wild-type DNA at a concentration of 10 fM.

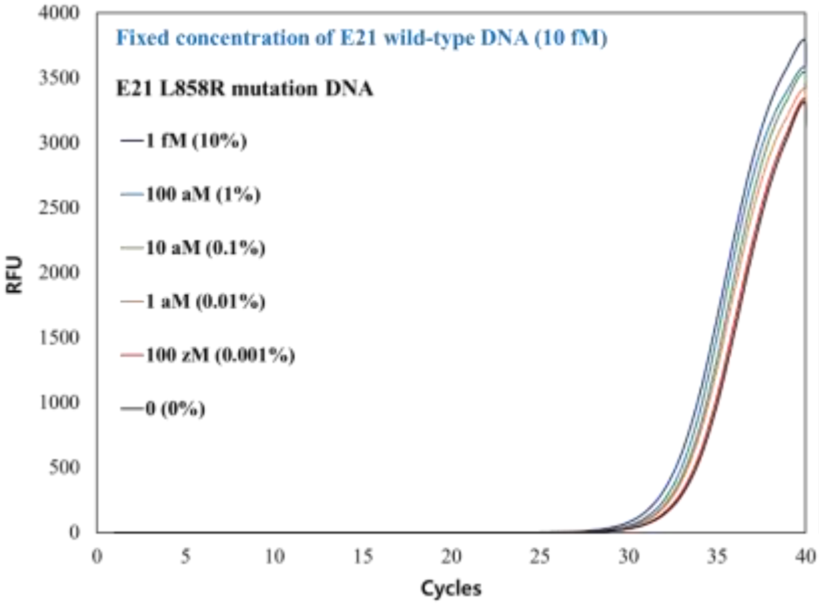

Wild-type DNA	E21 L858R mutation DNA	Ct value
10 fM	1 fM	32.6
	100 aM	32.98
	10 aM	33.19
	1 aM	33.29
	100 zM	33.65
	0	33.78

Figure S8. Recovery comparison of (A) our assay method and (B) real-time PCR, after cfDNA extraction using normal plasma samples (200 µL) spiked with EGFR E21 L858R DNA template at concentrations ranging from 100 fM to 100 zM.

(A)

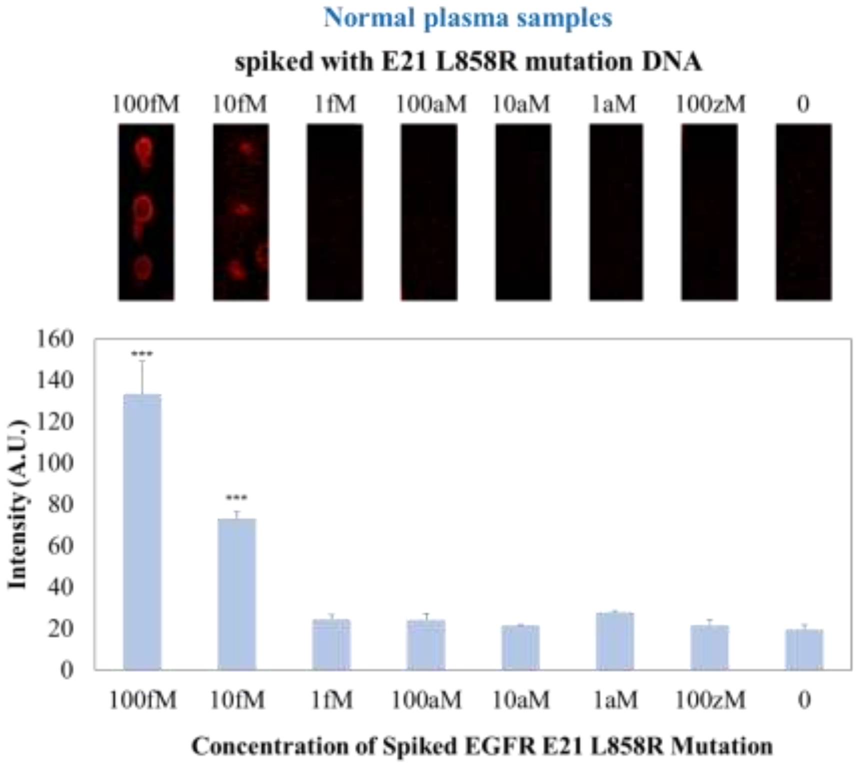

(B)

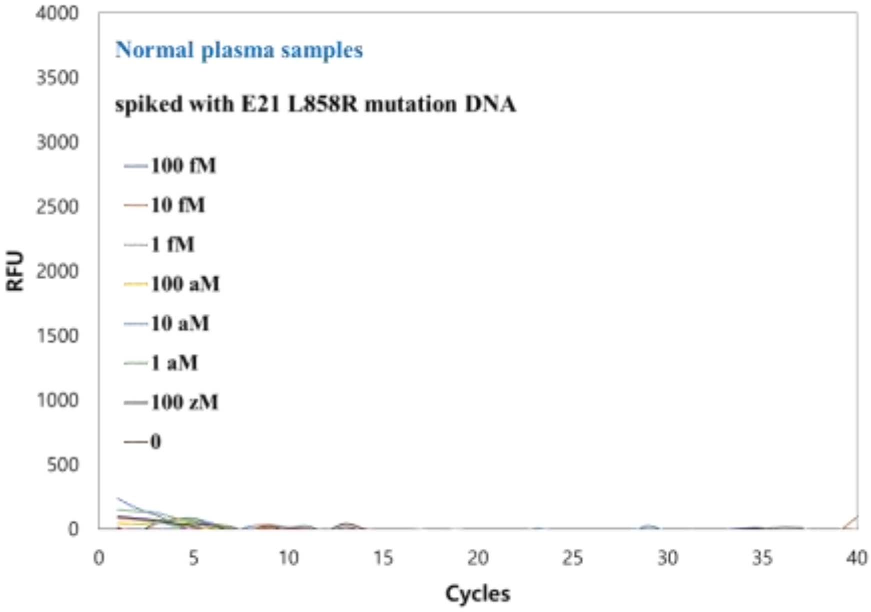

Figure S9. Photograph of the 3D nanoplasmonic-based EGFR mutation multiplex assay chip.

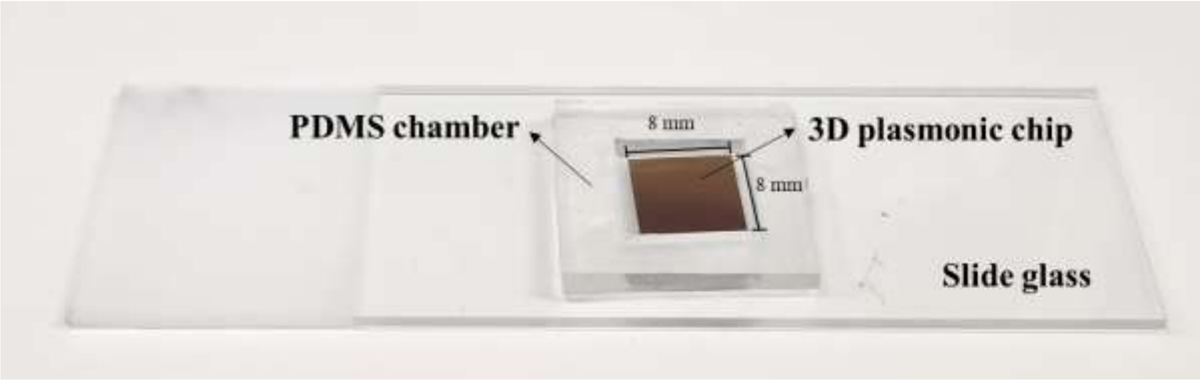

Table S1. Clinical characteristics of the patients with lung tumors involved in this study.

Classification	Patient	Age	Sex	Histological type	Cancer stage
Malignant lung tumor	#1	64	F	Adenocarcinoma	1A
	#2	70	M	Adenocarcinoma	1A
	#3	58	M	Squamous cell carcinoma	1A
	#4	68	M	Squamous cell carcinoma	2A
	#5	51	M	Adenocarcinoma	2B
	#6	64	M	Adenocarcinoma	2A
	#7	70	M	Squamous cell carcinoma	2B
	#8	50	F	Adenocarcinoma	3A
	#9	74	M	Small cell carcinoma	3A
	#10	63	M	Squamous cell carcinoma	3A
	#11	67	M	Adenocarcinoma	3A
	#12	56	F	Adenocarcinoma	3A
	#13	70	M	Squamous cell carcinoma	3A
	#14	67	M	Adenocarcinoma	3B
	#15	62	M	Small cell carcinoma	4A
	#16	66	F	Adenocarcinoma	4A
	#17	69	F	Adenocarcinoma	4A
	#18	45	M	Adenocarcinoma	4A
	#19	71	F	Adenocarcinoma	4A
	#20	50	F	Adenocarcinoma	4A
	#21	47	M	Adenocarcinoma	4B
Benign lung tumor	#22	58	M	-	
	#23	66	F		
	#24	67	M		
	#25	73	F		
	#26	73	F		
	#27	60	F		
	#28	69	F		
	#29	58	M		
	#30	69	M		
	#31	51	M		

Table S2. NGS panel of a 53-gene for whole-exome sequencing.

Mutation type	Gene									
**Point Mutation** [53]	ALK	BRAF	EGFR	ERBB2	IDH1	IDH2	KIT	KRAS	MYC	MYCN
	NRAS	PDGFRA	AKT1	APC	AR	ARAF	CCND1	CCND2	CCND3	CDK4
	CDK6	CHEK2	CTNNB1	DDR2	ERBB3	ERG	ESR1	ETV1	FBXW7	FGFR1
	FGFR2	FGFR3	FGFR4	FLT3	GNA11	GNAQ	GNAS	HRAS	MAP2K1	MAP2K2
	MET	MTOR	NTRK1	NTRK3	PIK3CA	PTEN	RAF1	RET	ROS1	SF3B1
	SMAD4	SMO	TP53							
**CNV** (Amplification gain /Insertion /Deletion) [13]	EGFR	ERBB2	MYC	MYCN	CCND1	CCND2	CCND3	CDK4	CDK6	FGFR1
	FGFR2	FGFR3	MET							
**RNA Fusion** [12]	ALK	BRAF	ERG	ETV1	FGFR1	FGFR2	FGFR3	MET	NTRK1	NTRK3
	RET	ROS1								

Table S3. NGS results for malignant lung tumor patients. Patients highlighted in blue letters have been identified with EGFR mutations.

Patient	Cancer stage	Mutated Gene	DNA sequence	Protein	Variant Allele Frequency (%)	Depth (X)
#1	1A	-	-	-	-	-
#2	1A	-	-	-	-	-
#3	1A	-	-	-	-	-
#4	2A	-	-	-	-	-
#5	2B	-	-	-	-	-
#6	2A	-	-	-	-	-
#7	2B	TP53	c.473G>A	p.R158H	0.6	1,629
#8	3A	EGFR	c.2236_2250del	p.E746_A750del	2.8	1,370
#9	3A	-	-	-	-	-
#10	3A	-	-	-	-	-
#11	3A	-	-	-	-	-
#12	3A	-	-	-	-	-
#13	3A	KRAS	c.35G>C	p.G12A	5.9	977
		TP53	c.854A>T	p.E285V	9.3	527
#14	3B	-	-	-	-	-
#15	4A	-	-	-	-	-
#16	4A	-	-	-	-	-
#17	4A	EGFR	c.2235-2249del	p.E746_A750del	1.3	4,786
		FBXW7	c.1393C>T	p.R465C	0.1	7,336
#18	4A	ERBB2	c.2313_2324dup	p.Y772_A775dup	6.7	1,621
#19	4A	EGFR	c.2573T>G	p.L858R	31	8,989
		TP53	c.853G>A	p.E285K	3	2,561
#20	4A	EGFR	c.2573T>G	p.L858R	25.1	7,520
			Amplification	1.5 copy number gain		
		TP53	c.586C>T	p.R196	5.8	4,335
			c.566C>T	p.A189V	53	4,335
#21	4B	EGFR	c.2573T>G	p.L858R	1.4	3,179
		TP53	c.814G>A	p.V272M	0.5	1,326
		MET	c.3334C>T	p.H1112Y	0.5	1,675

Figure S10. Plot of the fluorescence intensity of malignant lung tumor patients in Figure 5.

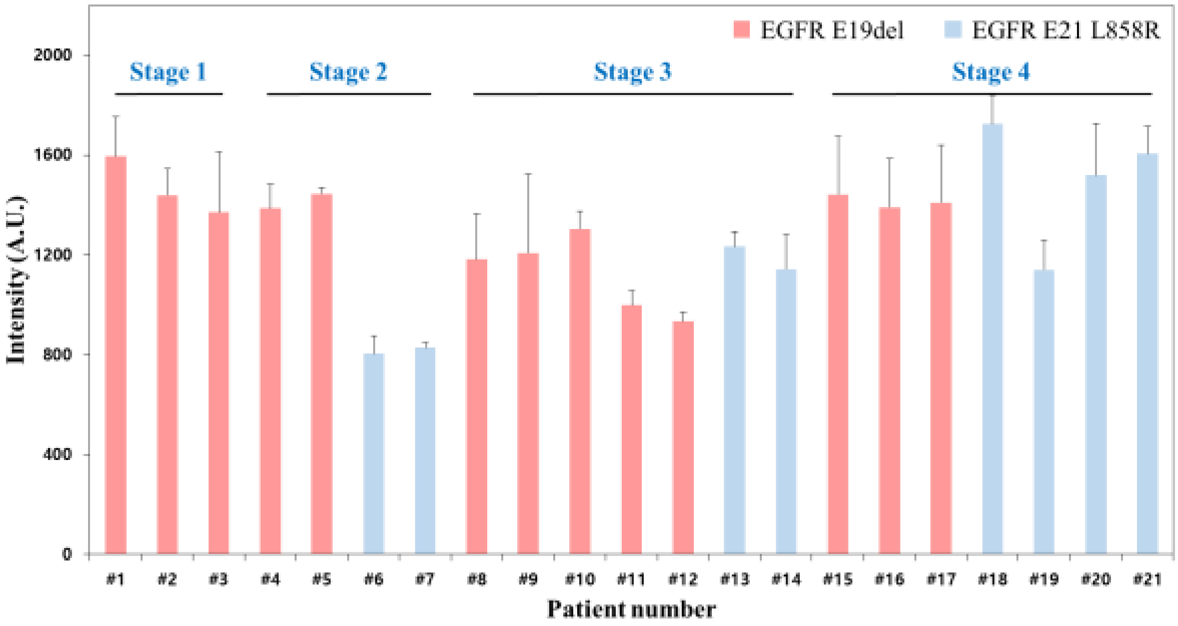

Supplement: Supplementary file 1 — Supplementary Material [file SMSC-4-2400101-s001.pdf]
